# Effectiveness of the RB51 vaccine in controlling brucellosis in adult beef cows

**DOI:** 10.3389/fvets.2024.1440599

**Published:** 2024-11-15

**Authors:** Carlos Antônio de Carvalho Fernandes, Gustavo Henrique Souza Pereira, Jessica Ruiz Pereira, Daniele Cristina Alves, Lucas Souza Dias, João Henrique Moreira Viana, João Drumond

**Affiliations:** ^1^Universidade Prof. Edson Antônio Velano, Alfenas, Minas Gerais, Brazil; ^2^Biotran Assessoria e Consultoria em Medicina Veterinária, Alfenas, Minas Gerais, Brazil; ^3^Embrapa Recursos Genéticos e Biotecnologia, Brasília, Distrito Federal, Brazil; ^4^MSD Animal Health, São Paulo, São Paulo, Brazil

**Keywords:** *Brucella abortus*, eradication, cattle, rough strain, zoonosis

## Abstract

**Introduction:**

The aim of this study was to compare the effectiveness of brucellosis control and reproductive performance using one or two doses of RB51 strain vaccine.

**Methods:**

This experiment was conducted at two commercial beef farms (A, *n* = 477; and B, *n* = 673), which were selected due to their records of endemic brucellosis and a prevalence of 6 to 8% of seropositive cows. An initial serology screening (Day 0) was carried out in all cows using the Rose Bengal test (RB), and presumptive positive results were re-evaluated using a slow agglutination test with 2-mercaptoethanol (2-ME). Seropositive cows (64, 5.6%) were culled. Non-reactive cows were then randomly assigned into three experimental groups: G1, a single vaccination with RB51; G2, two doses of RB51 given 6 months apart; and G3 (control group), no vaccination. Serological tests were carried out on Days 90, 180, 270, and 360. In each evaluation, seropositive cows were removed. The variables related to occurrence of new infections and abortion, as well as those related to subsequent reproductive performance, were analyzed using the SAS software.

**Results and discussion:**

Seropositive cows were still detected in both vaccinated and control groups at 90 days. However, no new infections were detected in G1 at 180 days or in G1 and G2 at 270 and 360 days, whereas new seropositive cows were diagnosed in all exams in G3 (control). Therefore, the cumulative number of new infections was lower in vaccinated than in control cows (0.2% and 0.0%, vs. 3.2% for groups G1, G2, and G3 respectively; *p* = 0.0002). In farm A the number of days open was greater in the control than in vaccinated groups (*p* < 0.05), and in farm B the pregnancy rate was lower in the control than in the group vaccinated once (*p* < 0.05). In summary, vaccination with RB51 in beef cows reduces the occurrence of new cases of brucellosis and improves the reproductive performance. There is no indication that a second immunization, six months after the first, enhances protection or reproductive efficiency.

## 1 Introduction

Brucellosis is a highly contagious disease caused by *Brucella abortus* (1). It is a zoonotic disease of global importance with a nearly worldwide distribution, as few countries have eradicated the disease. There are reports of high seroprevalence of *Brucella abortus*, particularly in developing or underdeveloped countries (2–8). It causes significant losses in cattle, primarily by increasing abortions and reducing the reproductive performance of herds (9). Brucellosis is rarely fatal in humans, but it can cause substantial morbidity that requires long-term treatment (9).

Due to the lack of vaccines for humans, the prevention of infections relies on controlling the disease in farm animals. In livestock, infection occurs mainly due to the ingestion of water or food contaminated with uterine discharges, placental debris, and aborted fetuses from infected animals, which may spread the bacteria for up to 30 days. Therefore, preventing the disease in pregnant cattle is essential to control the transmission of the pathogen (10).

Vaccinating young female calves and prepubertal heifers (aged 3–8 months) with the S19 strain is the standard procedure adopted for brucellosis control in cattle in Brazil. However, this strategy is not recommended for pubertal heifers and cows, as well as for male calves, as it induces the production of specific antibodies that interfere with serological diagnosis and may cause abortions in pregnant cattle (1). On the other hand, rough strain vaccines, such as RB51, do not induce the production of antibodies against lipopolysaccharide O (O-PS) and, therefore, do not interfere with conventional serologic diagnostic methods used to test adult cattle (11).

The RB51 vaccine has been successfully used in various programs to control brucellosis outbreaks through mass vaccination of herds in countries such as the USA (12), Chile (13), Azores (14), Spain (15), and Brazil (10). Vaccinating adult cows with the RB51 strain is among the strategies reported in brucellosis control programs to minimize the spread of the disease in commercial herds in endemic areas (16). However, whether a single vaccination dose is effective remains to be evaluated. Thus, this study aimed to compare the effectiveness of brucellosis control using one or two doses of RB51 (administered at a 6-month interval) in commercial beef farms previously vaccinated with strain 19.

## 2 Materials and methods

The experiment was carried out in two commercial beef farms in Minas Gerais, Brazil, from September 2020 to June 2022. These farms were selected because of their records of endemic brucellosis and a prevalence of 6–8% of seropositive cows. Both farms vaccinated only female calves, aged 3–8 months, with the S19 vaccine. During the experimental period, the cows were raised on a *Urochloa* sp. pasture with *ad libitum* access to a commercial mineral mixture. Farm 1 used fixed-time artificial insemination (FTAI), with inseminations and births occurring throughout the year. Farm 2 adopted a breeding season from December to March, with an initial FTAI followed by natural mating using seronegative bulls.

On day 0, an initial serology test was carried out using the buffered plate agglutination test (BPAT) or the Rose Bengal (RB) plate agglutination test on all cows, and those with presumptive positive results were re-evaluated using a slow agglutination test with 2-mercaptoethanol (2-ME). These tests are used to diagnose *B. abortus* in cattle (17), and the Brazilian National Program for the Control and Eradication of Brucellosis and Tuberculosis (PNCEBT) of the Brazilian Ministry of Agriculture, Livestock and Food Supply (MAPA) recommends them as standard tests for the screening and confirmation of brucellosis (18). Cows confirmed as seropositive by the 2-ME test were culled. The 2-ME test was used as a confirmatory test due to its high specificity (99.2%) (19). Non-reactive cows in each farm were randomly assigned to three treatment groups that were balanced for parity and Body Condition Score (BCS): G1 (*N* = 330), which received a single Subcutaneous (SC) vaccination dose in the neck region with RB51 (2 mL SC, lyophilized-reconstituted RB51^®^, MSD Saude Animal Brasil, São Paulo, Brazil; batch 007/20); G2 (*n* = 283), which received two SC vaccination doses of the RB51 vaccine, given 6 months apart; and G3 (*n* = 312, control group), which received no treatment. Some cows were removed from the experiment between days 0 and 90 (*n* = 161), between days 90 and 180 (*n* = 82), or between days 270 and 360 (*n* = 11) due to death or culling for unrelated reasons. Within each herd, cows from all three treatment groups were managed together, i.e., they had equivalent exposure to environmental contamination by *B. abortus*.

Serological surveillance was carried out using the RB test performed on days 90, 180, 270, and 360. All suspected positive outcomes in the RB test were checked with 2-ME, and cows confirmed as seropositive were culled. On both farms, we recorded data on abortions that occurred after vaccination.

Due to the differences in reproductive management between Farms 1 and 2 (adoption or non-adoption of the breeding season), different endpoints were considered to evaluate the effect of vaccination on the reproductive efficiency of each farm. On Farm 1, where FTAI was carried out throughout the year, we considered the interval between calving and the first AI, the number of AIs per conception, and the number of days open. On Farm 2, which adopted a breeding season with FTAI followed by natural mating, we could only calculate the final pregnancy rate.

The binomial variables (occurrence of new infections and abortion) were analyzed using a logistic regression procedure in SAS (SAS Institute Inc., Cary, NC), which included treatment effects, pregnancy days, parity, BCS, and their interactions. The variables of the interval between calving and the first AI, the number of AIs per conception, and days open were compared between groups using the analysis of variance (ANOVA) and PROC GLM, and the means were contrasted by performing Tukey’s *ad-hoc* test. The pregnancy rates were compared using a chi-squared test. Results are shown as mean ± SD or percentages. A *p*-value of <0.05 was considered to determine statistical significance.

## 3 Results

There was no significant difference (*p* > 0.05) in the average age (in years) of cows allocated to groups G1, G2, or G3 (5.4 ± 3.7, 5.8 ± 3.5, and 5.6 ± 3.3 for Farm 1 and 6.7 ± 3.5, 6.8 ± 3.3, and 6.7 ± 3.7 for Farm 2, respectively). The results of the initial serological test for brucellosis using RB and 2-ME tests are shown in Table 1. No difference (*p* > 0.05) was found in the prevalence of the disease between Farms 1 and 2.

**Table 1:** Outcomes of the first serological screening for brucellosis using Rose Bengal (RB) and 2-mercaptoethanol (2-ME) tests on the beef farms evaluated.

Farm	N	Presumptive seropositive (RB+)	Confirmed seropositive (2-ME+)
1	477	53 (11.1%)	26 (5.5%)
2	673	69 (10.3%)	38 (5.7%)
Combined	1,150	122 (10.6%)	64 (5.6%)

**Figure 1:**
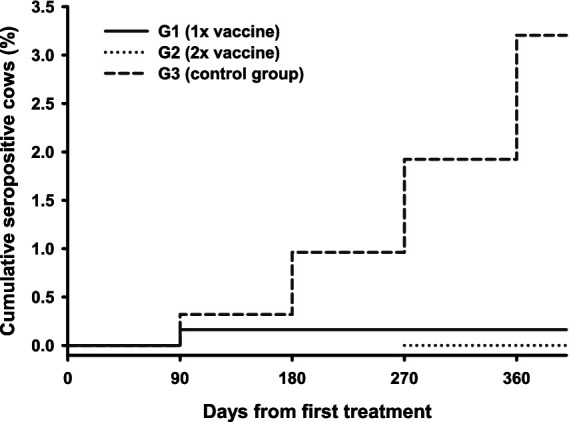
Cumulative percentage of cows infected after beginning the study.

The results of the serological surveillance performed on days 90, 180, 270, and 360 are shown in Table 2. Seropositive cows were still detected in both the vaccinated and control groups at 90 days (with 1 out of 613 and 1/312 for G1 and G3, respectively). However, no new seropositive cows were detected in G1 at 180 days or in G1 and G2 at 270 and 360 days, whereas new seropositive cows were detected in all examinations in G3 (control). Therefore, the cumulative number of new infections was lower in vaccinated cows than in control cows (0.2%^a^, 0.0%^a^, and 3.2%^b^ for groups G1, G2, and G3, respectively; *p* = 0.0002), as shown in Figure 1.

**Table 2:** Results of serological screening using RB and 2-ME tests obtained from beef cows on the two farms at 90, 180, 270, and 360 days after vaccination against brucellosis with RB51 once (G1), twice (G2), or not at all in the control group (G3).

Day^1^	Farm	*n* ^2^	Test^3^	G1 (1× vaccine)	G2 (2× vaccine)^4^	G3 (control)
90	1	404	RB	15/266	–	6/138
			2-ME	0/266	–	0/138
	2	521	RB	18/347	–	10/174
			2-ME	1/347	–	1/174
	Combined	925	RB	5.4%^a^ (33/613)	–	5.1%^a^ (16/312)
			2-ME	0.2%^a^ (1/613)	–	0.3%^a^ (1/312)
180	1	403	RB	13/265	–	4/138
			2-ME	0/265	–	1/138
	2	438	RB	13/295	–	5/143
			2-ME	0/295	–	1/143
	Combined	841	RB	4.6%^a^ (26/560)	–	3.2%^a^ (9/281)
			2-ME	0.0%^a^ (0/560)	–	0.7%^a^ (2/281)
270	1	402	RB	8/130	6/135	5/137
			2-ME	0/130	0/135	1/137
	2	437	RB	6/147	5/148	4/142
			2-ME	0/147	0/148	2/142
	Combined	839	RB	5.1%^a^ (14/277)	3.9%^a^ (11/283)	3.2%^a^ (9/279)
			2-ME	0.0%^a^ (0/277)	0.0%^a^ (0/283)	1.1%^a^ (3/279)
360	1	398	RB	7/128	6/135	5/135
			2-ME	0/128	0/135	2/135
	2	430	RB	6/146	5/144	4/139
			2-ME	0/146	0/144	2/139
	Combined	828	RB	4.7%^a^ (13/274)	3.9%^a^ (11/279)	3.3%^a^ (9/274)
			2-ME	0.0%^a^ (0/274)	0.0%^a^ (0/279)	1.5%^b^ (4/274)
	Accumulated^5^		2-ME	0.2%^a^ (1/613)	0.0%^a^ (0/283)	3.2%^b^ (10/312)

1 Days after the first vaccination.

2 Numbers decreased due to the culling of seropositive cows or for other unrelated reasons.

3 RB, Rose Bengal test; 2-ME, 2-mercaptoethanol test.

4 Data from G2 were only considered after the second vaccination dose, given on day 180.

5 Percentage of confirmed seropositive/original population.

a,b Percentages followed by different superscripts, on the same row, differ (chi-squared or Fisher’s exact tests, *p* < 0.05).

The occurrence of abortion after the vaccination is shown in Table 3. We observed no difference (*p* > 0.05) in the incidence of abortion among groups.

**Table 3:** Incidence of abortion in the beef cows after immunization against brucellosis with the RB51 vaccine once (G1), twice (G2), or not at all in the control group (G3).

Group		Abortion	
	Farm 1	Farm 2	Combined
G1 (1× vaccine)	1.7% (3/176)	2.7% (4/146)	2.2% (7/322)
G2 (2× vaccine)	3.0% (4/135)	2.1% (3/144)	2.5% (7/279)
G3 (Control)	4.3% (6/138)	5.0% (7/139)	4.7% (13/277)

Reproductive efficiency outcomes from Farm 1 are shown in Table 4. There was no significant difference (*p* > 0.05) in the interval from calving to the first AI, the number of AIs per conception, or the percentage of abortion. However, unvaccinated cows (G3) had a greater number of days open than vaccinated cows (G1 and G2).

**Table 4:** Reproductive efficiency of cows vaccinated once (G1) or twice (G2) with RB51, and those from the control group (non-RB51 vaccinated), on a beef farm that adopted FTAI throughout the year.

Group	*N*	Calving-first AI^1^	AI/conception	Days open
G1 (1× vaccine)	176	36.2 ± 7.8^a^	1.91 ± 0.24^a^	79.7 ± 14.2^b^
G2 (2× vaccine)	135	35.4 ± 7.2^a^	1.83 ± 0.22^a^	77.2 ± 13.7^b^
G3 (Control)	138	39.1 ± 8.9^a^	2.25 ± 0.4^a^	85.5 ± 15.9^a^

1 Means in days.

a,b Values followed by different superscripts, on the same column, differ (*p* < 0.05).

There was no difference (*p* > 0.05) in the interval from calving to the first AI or in the percentage of abortion among the groups. However, the pregnancy rate at the end of the breeding season was greater (*p* = 0.0376) in cows from G1 compared to G3, although neither were different (*p* > 0.05) from G2, as shown in Table 5.

**Table 5:** Reproductive efficiency of the cows vaccinated once (G1) or twice (G2) with RB51, as well as those from the control group (non-RB51 vaccinated), on a beef farm that adopted a breeding season.

Group	*N*	Calving-first AI^1^	Pregnancy rate
G1 (1× vaccine)	138	41.2 ± 9.6^a^	89.1%^a^
G2 (2× vaccine)	137	40.5 ± 10.2^a^	85.0%^ab^
G3 (Control)	141	43.1 ± 9.8^a^	80.1%^b^

1 Means in days.

a,b Values followed by different superscripts, on the same column, differ (*p* < 0.05).

## 4 Discussion

In the current study, we evaluated two strategies for controlling endemic brucellosis in beef cows by immunizing adult pregnant cows with the rough RB51 strain vaccine. We hypothesized that vaccination would control the occurrence of new cases within the herd without affecting the results of routine serological tests, such as the Rose Bengal (RB) test. Additionally, we hypothesized that a booster vaccination dose 180 days later would improve protection against infection. Our results supported our first hypothesis, as demonstrated by the lack of new seropositive cows within the vaccinated group from 180 days onward. However, there was no evidence to support the necessity of a booster vaccination dose (second RB51 booster dose) to ensure short-term protection against brucellosis.

The primary concerns regarding vaccination against brucellosis in adult cattle are the potential interference with routine serological tests, such as the RB test, observed with the S19 strain, and the possibility of inducing abortion in pregnant cows. In this regard, we observed no changes in the percentage of positive reactions in the RB test following the first or second vaccination dose with RB51, which indicates that this strategy would not interfere with brucellosis surveillance. If vaccination with strain RB51 were to increase seroconversion, which was not observed in the current study, it would result in a greater demand for more expensive confirmation tests, such as 2-ME. On the other hand, our results suggested that the seroprevalence reported by studies that used the RB test as the sole diagnostic test may have been overestimated, which led to the unnecessary culling of many false-positive cows.

In our study, we observed no difference in the percentage of abortion between the immunized (once or twice) and non-immunized cows. This result confirms the previous findings reported by Barbosa et al. (10), who demonstrated the safety of vaccinating cattle with RB51 at different stages of pregnancy.

In our post-treatment surveillance, one seropositive cow was detected 90 days after the vaccination. However, this was most likely due to testing of a recently infected cow, as no other seropositive cows were found in the vaccinated groups up to 360 days after the initial immunization, whereas new infections were observed in all subsequent tests in the control (non-vaccinated) group. As the cows from all groups were raised together, the observed difference indicates that the pathogen was present in the environment and vaccination with RB51 protected the cows from infection and prevented the development of the disease. In addition, it highlights the limitations of using culling as the sole strategy to control brucellosis. In the control group (G3), new infections were observed in all examinations, even though all the seropositive cows were culled after each examination. Few countries managed to eliminate brucellosis from endemic cattle herds only by culling seropositive animals without any complementary measures (16). Our results confirm this difficulty.

Our second hypothesis, i.e., the potential advantage of a booster dose 180 days after the first vaccination dose with RB51, was not supported by the results. Further research is required to evaluate whether a second dose of vaccine would be beneficial in herds with a higher prevalence of the disease, or to ensure long-term protection in places where the disease cannot be completely eradicated, for example, if present in wildlife.

Overall, the endpoints evaluated in the farms enrolled in the current study suggest an improvement in reproductive performance after the vaccination against brucellosis, despite the differences in the breeding systems adopted. The reproductive problems caused by brucellosis and their impact on herd reproductive performance are well known (20). In particular, abortion during the last trimester of pregnancy and the consequent high incidence of retained placenta are expected to have a more immediate impact on parameters such as the pregnancy rate or days open than on the interval from calving to the first AI or AI per conception, as observed in this study. However, it is likely that a statistically significant effect will also be observed on most fertility indexes if a greater amount of data were analyzed.

## 5 Conclusion

The immunization with a single dose of the rough strain RB51 vaccine effectively prevented new brucellosis infections in beef cows managed under extensive conditions without increasing the occurrence of false positives. Moreover, vaccination with RB51 improved the reproductive performance of herds with endemic brucellosis.

## Data Availability

The raw data supporting the conclusions of this article will be made available by the authors, without undue reservation.
